# Not Too Much and Not Too Little: Information Processing for a Good Purchase Decision

**DOI:** 10.3389/fpsyg.2021.642641

**Published:** 2021-04-28

**Authors:** Claudia Vogrincic-Haselbacher, Joachim I. Krueger, Brigitta Lurger, Isabelle Dinslaken, Julian Anslinger, Florian Caks, Arnd Florack, Hilmar Brohmer, Ursula Athenstaedt

**Affiliations:** ^1^Department of Psychology, University of Graz, Graz, Austria; ^2^Department of Cognitive, Linguistic and Psychological Sciences, Brown University, Providence, RI, United States; ^3^Institute of Civil Law, Foreign Private Law and Private International Law, University of Graz, Graz, Austria; ^4^Department of Psychology, Bundeswehr University Munich, Munich, Germany; ^5^Interdisciplinary Research Centre for Technology, Work and Culture, Graz, Austria; ^6^Development Bank of Austria, Vienna, Austria; ^7^Department of Applied Psychology, University of Vienna, Vienna, Austria

**Keywords:** decision making, information processing, decision quality, consumer segmentation, behavioral tracking

## Abstract

When deciding on an online purchase, consumers often face a plethora of information. Yet, individuals consumers differ greatly in the amount of information they are willing and able to acquire and process before making purchasing decisions. Extensively processing all available information does not necessarily promote good decisions. Instead, the empirical evidence suggests that reviewing too much information or too many choice alternatives can impair decision quality. Using simulated contract conclusion scenarios, we identify distinctive types of information processing styles and find that certain search and selection strategies predict the quality of the final choice. Participants (*N* = 363) chose a cellular service contract in a web-based environment that closely resembled actual online settings in the country of study. Using information processing data obtained with tracking software, we identify three consumer segments differing along two dimensions – the *extent* dimension, referring to the overall effort invested in information processing, and the *focus* dimension, referring to the degree to which someone focuses on the best available options. The three subgroups of respondents can be characterized as follows: (1) consumers with a low-effort and low-focus information processing strategy (*n* = 137); (2) consumers with a moderate-effort and high-focus information processing strategy (*n* = 124); and (3) consumers with high-effort and low-focus information processing strategy (*n* = 102). The three groups differed not only in their information processing but also in the quality of their decisions. In line with the assumption of ecological rationality, most successful search strategies were not exhaustive, but instead involved the focused selection and processing of a medium amount of information. Implications for effective consumer information are provided.

## Introduction

When deciding on a purchase, consumers often face a plethora of information. The rapid growth of e-commerce has paved the way to producing, retrieving, and distributing information ever more easily, faster, and cheaper (Lee and Lee, 2004). Vast amounts of rapidly changing options, along with information provided via multiple sources, have also created information overload and thereby challenges to rational choice (Lee and Cho, 2005). Finding judicious ways to control the flow of information has emerged as an important task (Zander and Hamm, 2012). Corporate objectives and sellers’ legal responsibilities contribute to this oversupply of information (Ben-Shahar and Schneider, 2011). The obligation to provide consumers with certain types of information, such as general terms and conditions, is meant to protect them from making decisions that may not meet their needs and interests. The psychological assumptions underlying these regulations are that consumers are willing and able to process large amounts of data about alternative courses of action, rank outcomes in order of expected utility, and choose that alternative which will yield the optimal outcome. These assumptions may be too strong. There is strong evidence, that people manage complex decision environments without maximizing utility. Everyday consumers rarely meet the standards of *homo economicus* (Bakos et al., 2009; Choi et al., 2010; Marotta-Wurgler and Chen, 2012; Becher and Unger-Aviram, 2015). Moreover, there is considerable variation in how consumers approach and implement their decision tasks when facing product choices (e.g., Delaney et al., 2015; Mittal, 2017). Consumers differ in the amount of information they are willing and able to acquire and process before making decisions (Mittal, 2017), and particularly so when important outcomes are at stake. Not every consumer benefits the same way from the availability of information online.

The present research focuses on identifying and exploring different types of information processing styles and how search and selection strategies predict the quality of the final purchase choice in a simulated online contract conclusion scenario. Unlike previous studies (e.g., Delaney et al., 2015; Mittal, 2017; Makgosa and Sangodoyin, 2018), we moved beyond self-report measures of information processing and measured actual information processing behavior by obtaining time- and frequency estimates with tracking software. By including objective criteria for choice quality, the present research illuminates the interplay of information processing behavior and choice quality in an applied online consumer context that has not been addressed in previous research. Addressing this need, the present study contributes to the literature on the development of “ergonomic” (i.e., psychologically realistic and effective) regulation of consumer information.

### Information Processing Style

Most attempts to investigate the influence of information processing on decision making assume a two types of psychological processes (De Dreu et al., 2000; Scholten et al., 2007). Although the labels used in various dual-process model vary (Evans, 2008), the shared assumption is that information can be processed within an autonomous and intuitive system or within a more reflective system using systematic modes of processing. The first system, often simply referred to as *System 1*, allows fast and effortless ways of processing that are not necessarily conscious. Much of heuristic reasoning is of this type. Heuristics are simple decision rules, such as schemas and expectations, that are learned through experience and stored in memory (Bohner et al., 1995; Chen et al., 1996). Intuitive information processing is thought to require only little cognitive capacity. By contrast, the second system *(System 2)* requires analytical, comprehensive, rule-based, and deliberate ways of processing, where individuals seek to incorporate all available information in order to make optimal decisions. Systematic processing requires considerable effort and is more likely to occur when both cognitive capacity and motivation are at sufficient levels (Chen et al., 1999; Zuckerman and Chaiken, 1998; Chaiken et al., 1989).

Arguably, models limited to two modes of processing will not capture the full complexity of decision making (Keren and Schul, 2009; Delaney et al., 2015; Melnikoff and Bargh, 2018). In consumer research, other attempts to segment consumers have emerged. Sproles and Kendall (1986) proposed a multi-faceted taxonomy of consumer decision-making styles comprising: (i) perfectionist, high quality consumers, (ii) brand conscious, price equals quality consumers; (iii) novelty-fashion conscious consumers; (iv) recreational and hedonistic shopping conscious consumers; (v) price-conscious, value for money consumers; (vi) impulsive, careless consumers; (vii) confused by over-choice consumers; and (viii) habitual, brand-loyal consumers. Mittal (2017) distinguished between the extent (extending vs. info-mising) and the goal of information processing (optimizing vs. satisficing). On this view, some consumers, the extenders, are willing and able review large amounts of information, whereas others, the misers, process as little information as possible (misers). Similarly, optimizers try to make the best choice, whereas satisficers are willing to accept options if certain minimum requirements are met. Consumers also vary in their degree of indecision, that is, the difficulty they experience when making decisions. Using this trait model, Mittal (2017) identified four distinctive types of consumers: *optimizing extenders*, who seek the best option and are willing to undertake extensive information processing, *balanced and diligent consumers*, who consider information at hand but without being too extensive, *confused and uncertain foot draggers*, who do not optimize but exert some effort, and *snap deciders* who make gut decisions with minimal information processing effort. Makgosa and Sangodoyin (2018) identified three types of shoppers across a wide age range, being time/energy conserving, perfectionism, and habitual buying. Thus, depending on the context, sample, and strategy of analysis, studies differ on the exact number of segments identified. However, when considering the structural meaning of the segments, there is a considerable amount of overlap across the many studies that have been carried out on decision-making styles.

### Information Processing Style and Decision Quality

The conventional view in the social sciences is that deliberate information processing is vital for making sound decisions and that it will increase the probability of making the best decision. The idea that carefully processing all available information outperforms less deliberate processing is central to many classical and contemporary perspectives on decision making (Chaiken et al., 1989). Decision biases and errors are thought to arise from limited information processing (Tversky and Kahneman, 1974; Kahneman and Frederick, 2005). Yet, evidence shows that less systematic decision making often performs rather well (Simon, 1992; Gigerenzer, 1996; Gigerenzer and Brighton, 2009). Deliberately processing all available information does not necessarily yield the best decisions. Depending on the situation, individuals often reach good decisions by using simple processing strategies such as fast and frugal heuristics. Accordingly, several studies show an inverse-U-shaped relationship between level of accuracy and information processing efforts, in terms of amount of information considered, time spent on information processing, and computation invested (Gigerenzer and Brighton, 2009). Less systematic or intuitive strategies may yield excellent results when uncertainty is moderate or high (Hogarth and Karelaia, 2007; Rieskamp and Hoffrage, 2008), when the acquisition of information is costly (Bröder, 2000), when time is scarce (Ordonez and Benson, 1997; De Dreu, 2003), and, most importantly, when information load is high (Jacoby et al., 1974; Swain and Haka, 2000; Kasper et al., 2010). Within the context of (consumer) choice, Sasaki et al. (2011) showed that consumers who were confronted with product information that exceeded their processing capacity simply chose the product that enjoyed the highest popularity. Kasper et al. (2010) reported that participants switched to simple strategies when they felt overstrained by the available information. Such strategies included, for example, restricting information search to a specific provider, store, price or brand, or restricting attention to brand or price information. Another study found evidence that individuals who dealt intensively with a certain choice alternative, were less satisfied with their decision and regretted the waiver of the unelected alternative stronger, although they gathered more information and processed it more intensely, compared with individuals who based their decision on simple heuristics (Carmon et al., 2003).

It is generally acknowledged that humans have limited information processing capacity and, thus, are simply not able to fully process all available information in a high-load situation. To successfully cope with situations of high information load and to keep decision quality at acceptable levels, individuals need to apply different shortcut strategies (Kasper et al., 2010). Thus, consumers facing a complex contract decision may benefit from moderate, but not exhaustive information processing. However, the link between type of information processing and decision quality is still not fully understood. Although many studies have been carried out on information processing styles (e.g., Walsh et al., 2007; Kasper et al., 2010), studies on the relationship between information processing style and decision quality are lacking. Most studies focus on buying personal goods offline, and none of them focuses on online shopping and the special conditions consumer face online. Consumer taxonomies are typically based on self-report measures. There are, to the best of our knowledge, no studies that use actual information processing data obtained with behavioral tracking methods to segment consumers. Given the complexity and heterogeneity of previous research and the novelty of our own study design, we took an exploratory rather than strictly hypothesis-testing approach. We sought to contribute to the knowledge base by posing the following research questions:

RQ 1: Which consumer segments can be identified in a simulated online purchase when using actual information processing data obtained with tracking software?
RQ2: How do distinctive consumer segments differ in their average decision quality?

## Methods

### Sample

We recruited 371 participants and excluded the data of eight because of technical errors involving the tracking software or the online environment. The effective sample comprised 363 participants (214 females, 149 males), ranging in age from 18 to 71 years (*M* = 27.51, *SD* = 9.87). Since contract decisions, particularly for cellular services, affect the general population, we enrolled participants from diverse socio-economic and demographic backgrounds, using a variety of recruitment channels, such as social networks (21%), mailing lists (57%), as well as flyer and advertisements in print and broadcasting media (8%). After completing the study, each participant received €20 and a raffle ticket for a tablet computer.

### Decision-Making Task and Procedure

The procedure consisted of an online pre-task questionnaire, the main decision task, and a post-task questionnaire on decision-related issues. After registering online, participants completed a questionnaire on demographic data (LimeSurvey Project Team and Schmitz, 2015). Participants then received an identification code and were scheduled for the second part of the study.

The main part of the study was conducted in group sessions at a university computer lab. After arriving, participants entered their identification code. They were then asked to select the cellular service contract they found most appropriate from a set of different options. The cellular service market was chosen as the context for this study for the following reasons. First, this market is economically significant in EU Member States and the United States (Bar-Gill and Stone, 2009; Kasper et al., 2010; Bar-Gill, 2012). To illustrate, in the country of study, every person (including legal entities) is holding one and a half cellular service contracts on average. Similarly, there were 398 million cellular service subscribers by the end of 2016 in the United States market, implying a penetration rate of 121% (Federal Communications Commission [FCC], 2017). Second, the cellular service market is one of the most dynamic and turbulent markets in the world today (Turnbull et al., 2000). Since its deregulation, it has seen a rising number of service providers and contract options. At the same time, it has been assumed that market deregulation would allow consumers to choose the plans most beneficial to them. Yet, consumers often fail to do this (Lunn, 2013). Third, in telecommunication the total cost of the service typically has a multipart structure with different rates, modes of calculation, amounts of services, and contract clauses. Finally, the rapid technological development and innovation, which is typical of the cellular service market, adds further complexity, thus creating further risks of suboptimal contract choices (Lambrecht and Skiera, 2006; Bar-Gill and Stone, 2009; Gerpott, 2009; Grubb, 2009).

Participants were presented with the available information and asked to consider it in the way they would if they were actually choosing a cellular service contract. They were also asked to base their decision on a particular pattern of consumption (user profile) for a period of two years. In order to avoid any coincidence with the participants’ actual consumption behavior, they were randomly assigned to one of two different user profiles. Each profile contained information about a certain average monthly consumption of voice call minutes, text messages and internet data transfer (MB). The two profiles (profile 1: 1696 voice call minutes, 374 text messages and 980 MB data; profile 2: 1909 voice call minutes, 92 text messages and 2890 MB data) were constructed to ensure that results were not limited to a specific pattern of consumption. Though the offers and information presented reflected natural conditions, we chose the user-profile in a way that minimized the influence of previous experience and prevented participants from drawing conclusions not based on the provided information. For instance, the most popular provider was not among the optimal options, given the pattern of consumption the participants had to consider. As expected, there were no significant differences between the two profiles for any of the dependent measures (all *p* > 0.05). Therefore, data were collapsed over the two profiles in all subsequent analyses.

After receiving information about the user profile, participants were directed to a web-platform with hyperlinks to all cellular service contract options available (see Supplementary Figures 1, 2^1^). Links were presented in randomized order within a sidebar on the left side of the screen. By clicking on any of the links provided, participants could browse through the information about the corresponding offer. Each offer was presented in a way similar to their actual appearance on the web. We applied the design of the real web pages of the providers, including a large part of their present costs, information and website design. Minor changes were necessary for practical reasons (e.g., links to irrelevant pages, such as ads and bundle offers were removed). To represent the diversity of the cellular service market, most options available on cellular service market at the time of study preparation in the country of study, except business contract options and bundle offers, were shown. The 48 different contract options, from 11 cellular service providers, presented on 24 different pages, reflected the natural conditions regarding the level of choice and information load. The upper right corner of the screen showed the time remaining for the completion of the task. Based on a pretest, participants were allowed up to 40 minutes to indicate a decision. After making their choice, participants received a virtual shopping basket guiding them through a typical web-based contract conclusion procedure, including the possibility to displaying and accepting the general terms and conditions (GTC).

At the conclusion of the study, participants were directed to a questionnaire with post-decisional measures (i.e., subjective relevance of contract characteristics, epistemic motivation, involvement, decision difficulty, time pressure, satisfaction with the decision, control variables; see measures section for the details). Participants were then fully debriefed, paid, and thanked for their participation.

### Measures

#### Information Processing Parameters

Based on previous research (De Dreu, 2003; Lee and Cho, 2005; Rieskamp and Hoffrage, 2008; Paul and Nazareth, 2010) information processing was indexed along two dimensions: The *extent* dimension, referring to the overall effort someone puts into the information processing, and the *focus* dimension, referring to the degree to which a participant focuses on the best available options. We used the total number of clicks and the total time spent on information processing as proxies for the overall information processing *extent* (De Dreu, 2003; Lee and Cho, 2005; Paul and Nazareth, 2010). To measure *focus* of information processing, we used the proportion of clicks and time participants spent on pages of the three best contract options (*clicks on 3 best, time on 3 best*) (Rieskamp and Hoffrage, 2008). We also computed a *comparison index* from the sum of direct alternations between the pages of the best three options to reflect the rigor of search. Given that the best three options were similar to one another, we expected participants to compare them more extensively and systematically.

To get a subjective measure of what pieces of contract information were perceived to be of greatest relevance for the decision at hand, participants were asked to indicate the *subjective relevance of 25 different contract characteristics* (e.g., monthly charge, conditions of contract termination) on a 6-point-scale ranging from 1 (not important) to 6 (very important). Based on the results of a factor analysis (see Supplementary material), subjective relevance ratings were grouped into four decision factors - *substantial contract details* (e.g., total costs or monthly charge of the plan), *commitment*/*flexibility* (e.g., conditions of contract termination), *personal experience* (e.g., personal experience with excluded providers, gut feeling), and *secondary tariff details* (e.g., technical availability).

### Decision Quality

The objective quality of the chosen contract option was determined normatively (Lee and Lee, 2004) based on the average monthly costs represented by a range of expenses, such as the monthly charge, activation fee, overage costs, and service fees or discounts. Monthly costs were chosen as an indicator for decision quality because they match the central goal of present EU and national consumer protection regulation. According to this legislation, a choice should allow consumers to realize their individual wishes and preferences while representing a good price/quality ratio that does not violate their interests by inflicting pecuniary losses. However, consumer regulation goes beyond monetary losses and not all aspects that may play a role when actually deciding on a cellular service contract, such as, for instance, subjective preferences or net coverage, could be considered as an indicator for decision quality. Nonetheless, we assume that substantial financial losses represent a major harm to consumers.

This normative index was calculated for a period of two years. Thus, based on the pattern of consumption provided via the user profile, each decision alternative was associated with a certain amount of up-front costs, which was then subtracted from the costs of the optimal decision for each participant. It is important to note that the user profiles were chosen in a way that allowed us to determine an optimal decision and that enabled the *post hoc* identification of meaningful cost categories. We used these categorizations to avoid a bias due to excessively costly, and thus wrong choices. Hence, average monthly additional costs were treated as an ordinal variable with four levels: (1) choices at least €10 more expensive than the optimal choice; (2) choices between €5 and €10 more expensive than the optimal choice; (3) choices up to a maximum of €5 more expensive than the optimal choice; and (4) optimal choices, including the best three contracts with identical costs. Higher scores on this variable reflect lower monthly additional costs and, thus, a higher quality of the contract.

### Additional Psychological Variables

Unless noted otherwise, all ratings were made on 6-point scales (1 = does not apply at all, 6 = applies completely). Following earlier research (De Dreu et al., 2000; De Dreu, 2003), we measured *epistemic motivation* with three items: (a) “I tried to take into consideration all possible alternatives”, (b) “I tried to process information as thorough as possible”, and (c) “I thought deeply before making a decision”. Items were collapsed into a single scale of epistemic motivation (α = 0.73). Participants’ *involvement in the decision* was measured with six questions (e.g., “How much do you identify with the decision?”) and collapsed into a single scale of involvement (α = 0.73, see also Fischer et al., 2013). Perceived *decision difficulty* was measured with a single-item (*“Working on the task was difficult to me”*). *Time pressure* was measured with five items (De Dreu, 2003; Rieskamp and Hoffrage, 2008) and collapsed into a single scale (α = 0.89). *Satisfaction with the decision* was measured with six items and collapsed into a single scale (α = 0.76). Table 1 presents the survey items and their descriptive statistics.

**TABLE 1:** Variables and Summary Statistics.

**Measures**	**M**	**SD**
**Information processing**		
Extent		
Total clicks	61.5	26.4
Total time	1177.2	589.1
Focus		
Clicks on 3 best	0.20	0.10
Time on 3 best	0.20	0.10
Comparison index	5.10	5.50
Decision quality	3.16	1.12
Satisfaction with decision	5.06	0.74
I feel good with my decision.	5.17	0.90
I am satisfied with my decision.	5.26	0.80
I feel, I have made a good decision.	5.13	0.95
I feel, I have chosen the cheapest plan.	4.75	1.28
I feel, I had sufficient time to make my decision.	5.02	1.41
I feel, I had sufficient information to make my decision.	5.03	1.12
Cronbach’s α = 0.76		
Epistemic motivation	5.04	0.86
I tried to take into consideration all possible alternatives.	5.16	1.11
I tried to process information as thorough as possible.	4.94	1.05
I thought deeply before making a decision.	5.01	1.05
Cronbach’s α = 0.73		
Involvement	4.45	0.68
How much do you identify with the decision?	4.65	1.22
How confident are you that you have made a good choice?	5.06	0.85
Would you revise your decision if possible? (R)	4.59	1.22
How competent do you feel for making such decisions?	4.54	1.14
Do you think you could justify your decision adequately?	5.06	0.97
To what extent do you commit yourself to the decision?”	5.29	0.83
Cronbach’s α = 0.73		
Time pressure	2.16	1.15
I experienced the task as stressful.	2.15	1.36
If I had had more time, I would have considered more information.	2.62	1.81
I felt to be under pressure while making my decision.	2.21	1.56
Time left was an issue of concern during decision making.	1.77	1.25
I had sufficient time to think about my decision. (R)	2.18	1.51
Cronbach’s α = 0.89		
Decision difficulty		
Working on the task was difficult to me.	2.02	1.21

*Note: Items were answered on a six-point scale ranging from 1 (applies not at all) to 6 (applies completely). Items marked with an *(R)* are reverse coded.*

### Control Variables

We considered participants’ familiarity with contracting online in general and with cellular service contracts in particular, their familiarity with different activities in the internet (e.g., searching for information, social networks), their own cellular service consumption behavior, their knowledge of the cellular service market, and several demographic variables (e.g., age, gender, income, education). Because none of these variables showed any significant association with any of the measures of theoretical interest (all *p* > 0.05 in preliminary analyses), we did not consider them further.

## Results

First, we sought to identify distinctive groups of participants by submitting the data on individual information processing behavior to an explorative cluster analysis. Second, we asked how these clusters of individual information processing behavior are related to decision quality. Third, we asked whether individual-differences in epistemic motivation, involvement with the decision, perceived decision difficulty, perceived time pressure, and satisfaction with the decision are related to these clusters. All analyses were conducted using IBM SPSS, Version 25 (International Business Machines Corporation [IBM Corp], 2017), and visualizations were created using the packages ”GGaly” (Schloerke, 2021) and ”ggplot2” (Wickham, 2016) in R (R Core Team, 2019).

### General Results

Among the 48 available contract options, there were 33 with different monthly costs (not all available options were chosen; some options were of identical costs). The monthly costs in this sample ranged from €14.90 (optimal) to €3,945.96 (excessive). When ranked, these costs yielded a proxy variable for decision quality. A majority (57%) of the participants made optimal decisions (*N* = 207) by choosing one of the three best cellular service contracts (in terms of monthly costs) that were available for their respective user profile. However, 17.4% chose an option that was up to €5 more expensive, 10.5% chose a contract that was between €5 and €10 more expensive, and 15.2% chose a contract that was more than €10 more expensive than one of the optimal contracts. Table 1 in the supplementary materials displays the chosen options in rank-order (lower ranks represents lower costs, and thus higher decision quality) and the associated absolute monthly costs.

### Patterns of Information Processing

To identify groups of participants with similar patterns of information processing behavior, a cluster analysis was performed using the information processing variables of total clicks, total time, clicks and time on 3 best, and the comparison index, which were obtained with the tracking software. Before clustering, variables were standardized to control for unequal scaling of the variables. This transformation allows ready comparisons of the distributions of values across variables and makes values independent of the unit of measurement. Segmentation was then performed following a two-fold standard procedure suggested by Malhotra (2010); see also Wiedenbeck and Züll (2010). First, we performed a hierarchical cluster analysis using Ward’s method based on squared Euclidian distances to identify the number of clusters appropriate for the variables in the analysis. Second, we performed a k-means cluster analysis with a fixed number of clusters. The stability of the final cluster solution was verified based on (a) the visual inspection of the mean distances between clusters as depicted in the dendrogram (see Supplementary Material, Supplementary Figure 3), (b) the review of the distance coefficients in the agglomeration schedule, (c) the mean differences between clusters in the behavioral information processing parameters, as indicated by statistically significant *F-*values in the ANOVA’s, and (d) considerations on the structural meaning of the clusters (Loibl et al., 2009). Finally, we performed a discriminant function analysis to confirm predicted cluster membership.

**FIGURE 1:**
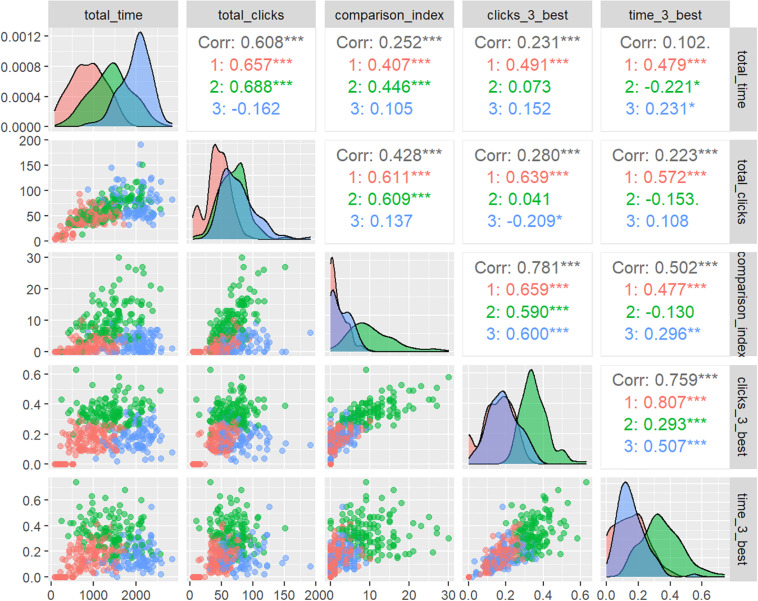
Pairs plot of cluster variables. Cluster 1 (red): low effort - low focus, cluster 2 (green): moderate effort - high focus, cluster 3 (blue): high effort - low focus; lower left: scatter plots split by clusters; diagonals: density plots comparing clusters; upper right: correlation coefficients per cluster, where *p < 0.05, **p < 0.01, ***p < 0.001.

The hierarchical cluster analysis, which was performed first, yielded a three-cluster solution, as depicted in the dendrogram. Review of the coefficients in the agglomeration schedule showed increased distance coefficients from case 360, also indicating a three-cluster solution. Follow-up K-means analysis yielded three clusters of similar sizes. Each of the five behavioral measures of information processing entered into the cluster analysis contributed significantly to the segmentation (total clicks: *F*(2, 360) = 105.79, *p* < 0.0001; total time: *F*(2, 360) = 220.58, *p* < 0.0001; clicks on 3 best: *F*(2, 360) = 252.48, *p* < 0.0001; time on 3 best: *F*(2, 360) = 140.1, *p* < 0.0001; comparison index: *F*(2, 360) = 224.72, *p* < 0.0001). The final grouping of cases into three clusters was determined after nine iterations of the k-means algorithm, as indicated by a zero-change in cluster centers after the ninth iteration. Figure 1 visualizes the standardized scores of cluster variables separated by cluster membership using pairs plots of cluster variables.

**FIGURE 2:**
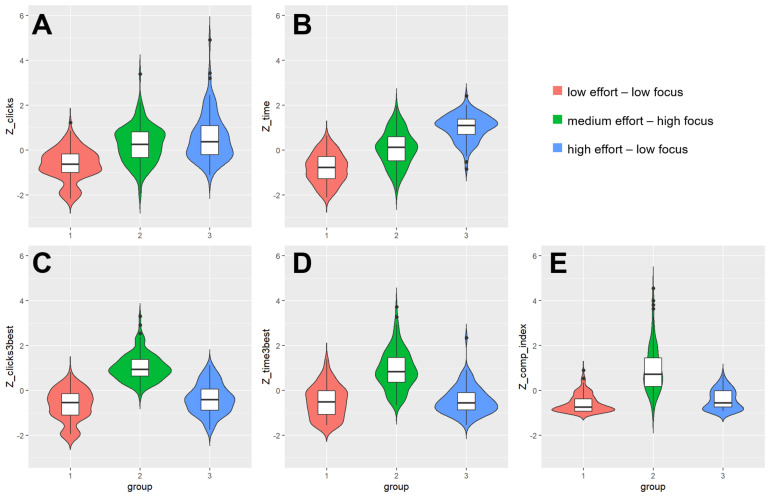
Box and violin plots of the five behavioral measures of information processing behavior as a function of cluster assignment regarding **(A)** number of clicks; **(B)** total time; **(C)** clicks on 3 best; **(D)** time on 3 best; and **(E)** comparison index. Note: Boxes depict interquartile range with median in the center; density curves depict the distribution of the cases.

To test the robustness of the solution, one-way analyses of variance (ANOVA) were computed on each of the five information processing measures and cluster membership as the independent variable. The ANOVA’s indicated that the three clusters significantly differed across each of the five information processing measures, total clicks, *F*(2, 360) = 67.88, *p* < 0.001, $\eta_{p}^{2}$ = 0.274, total time, *F*(2, 360) = 212.43, *p* < 0.001, $\eta_{p}^{2}$ = 0.541, clicks on 3 best, *F*(2, 360) = 236.22, *p* < 0.001, $\eta_{p}^{2}$ = 0.568, time on 3 best, *F*(2, 360) = 141.62, *p* < 0.001, $\eta_{p}^{2}$ = 0.440, and comparison index, *F*(2, 360) = 181.37, *p* < 0.001, $\eta_{p}^{2}$ = 0.502. Table 2 shows the results of these comparisons and the descriptive statistics. Figure 2 displays the cluster differences on the behavioral information processing parameters.

**TABLE 2:** Means, Standard Deviations and results of the ANOVA’s on the behavioral measures of information processing.

**Measure**	**Cluster 1 (*n* = 137)**		**Cluster 2 (*n* = 124)**		**Cluster 3 (*n* = 102)**		***F***	***p***	**$\eta_{p}^{2}$**
	***M***	***SD***	***M***	***SD***	***M***	***SD***			
Total clicks	44.3^c^	18.8	68.3^b^	22.1	76.4^a^	27.3	67.88	0.000	0.274
Total time	872.3^c^	40.6	1408.7^b^	472.9	1989.1^a^	654.6	212.43	0.000	0.541
Clicks on 3 best	0.15^c^	0.08	0.35^a^	0.07	0.19^b^	0.08	236.22	0.000	0.568
Time on 3 best	0.15^b^	0.10	0.35^a^	0.12	0.16^b^	0.09	141.62	0.000	0.440
Compariso*n* index	1.91^b^	2.16	10.44^a^	5.84	2.78^b^	2.44	181.37	0.000	0.502

*Superscript letters indicate results of pairwise comparisons, with same letters indicating non-significant differences based on *p* < 0.05.*

*Post hoc* comparisons with Bonferroni adjustments for multiple comparisons (see Table 2) indicated that participants in cluster 1 (low effort-low focus; *N* = 137) showed the least effortful information processing behavior, as indicated by the lower total clicks (*M* = 44.3, *SD* = 18.8) and less total viewing time (*M* = 872.3, *SD* = 40.6). Additionally, they exhibited a less focused information processing as indicated by a small number of clicks (*M* = 0.15, *SD* = 0.08) and less time spent (*M* = 0.15, *SD* = 0.10) on the three best alternatives and a low comparison index (*M* = 1.91, *SD* = 2.16). Participants in cluster 2 (moderate effort-high focus; *N* = 124) showed an intermediate degree of information processing effort, as suggested by the intermediate scores on the extent measures, total clicks (*M* = 68.3, *SD* = 22.1) and total time (*M* = 1408.7, *SD* = 472.9). At the same time, the focus of information processing, as indicated by the portion of clicks (*M* = 0.35, *SD* = 0.07) and portion of time spent (*M* = 0.35, *SD* = 0.12) on the three best alternatives as well as the comparison index (*M* = 10.44, *SD* = 5.84), was higher in this cluster than in the others. Participants in cluster 3 (high effort-low focus; *N* = 102) put the most effort into information processing, as indicated by a high number of total clicks (*M* = 76.4, SD = 27.3) and time (*M* = 1989.1, *SD* = 654.6). However, cluster 3 showed, similar to cluster 1, a less focused information search as indicated by a small portion of clicks (*M* = 0.19, *SD* = 0.08) and time spent (*M* = 0.16, *SD* = 0.09) on the three best alternatives as well as a low comparison index (*M* = 2.78, *SD* = 2.44).

A discriminant function analysis estimated the extent to which cases were correctly classified into clusters. This analysis involved a two-step procedure that, first, uses predictor variables to calculate discriminant functions to predict known group membership, and, second, calculates the percentage of cases that were correctly reclassified back into the original categories. In the present analysis, information processing variables originally entered into the cluster analysis (i.e., total clicks, total time, portion of clicks and time, comparison index) were used to predict known group membership (i.e., cluster membership).

Together, two discriminant functions accounted for 100% of the variance, with each function capturing a substantial part of the variance (see Table 3).

**TABLE 3:** Discriminant Functions.

**Function**	**Eigen value**	**% of variance**	**Effect size**	**Wilks’ Lambda**	**Chi-Square**	**df**	***p***
1	2.127	63.4	0.681	0.143	695.27	10	0.000
2	1.230	36.6	0.552	0.448	287.16	4	0.000

Based on the structure matrix (see Table 4), variables associated with the first discriminant function included high levels on the focus measures, that is, the proportion of clicks and time on three best alternatives and comparison index. Variables in the second discriminant function included high levels on the extent measures, that is, the total clicks and total time. Among all variables in the model, standardized canonical discriminant function coefficients indicated that total time, portion of time, and comparison index were more relevant in determining group membership (see Table 4). Overall, the results indicate a high degree of accuracy for all clusters, with 92% correctly identified cases. More specifically, 89.9% (cluster 1), 91.9% (cluster 2), and 95.1% (cluster 3) of the original grouped cases were correctly classified into the original clusters.

**TABLE 4:** Structure Matrix and Standardized Canonical Coefficients.

	**Structure matrix**		**Standardized canonical coefficients**	
	**Function**		**Function**	
	**1**	**2**	**1**	**2**
Total clicks	–0.043	0.551	–0.262	0.221
Total time	–0.290	0.902	–0.559	0.742
Portion clicks	0.642	0.595	0.105	0.404
Portion time	0.536	0.377	0.529	0.053
Comparison index	0.586	0.476	0.813	–0.108

**FIGURE 3:**
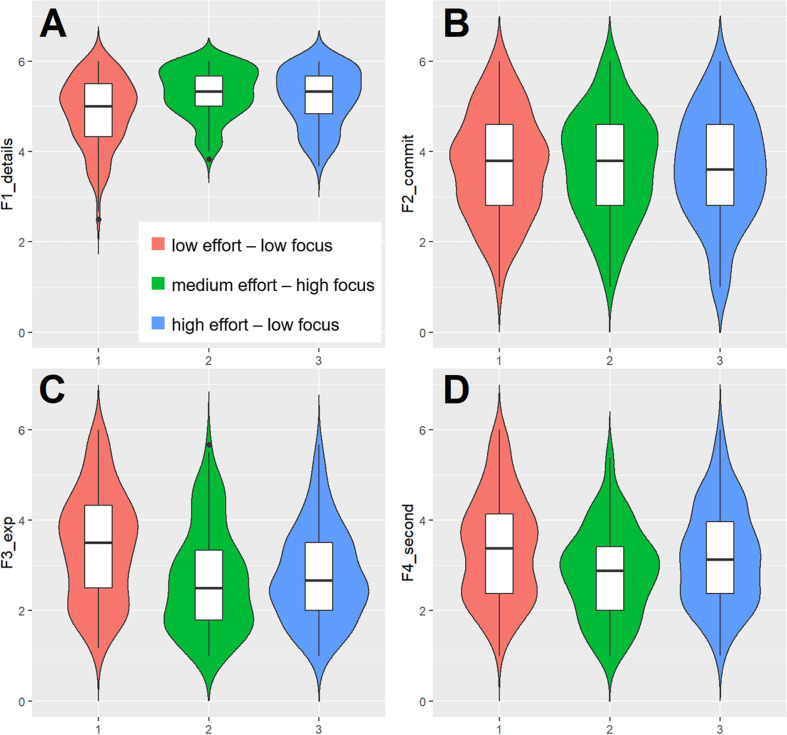
Box and violin plots of the subjective relevance ratings of the four decision factors as a function of cluster assignment regarding **(A)** substantial contract details; **(B)** commitment/flexibility; **(C)** personal experience; and **(D)** secondary contract details. Note: Boxes depict interquartile range with median in the center; density curves depict the distribution of the cases.

Finally, we were interested in whether the identified clusters differed on the perceived relevance associated with different contract characteristics. To this end, we computed an ANOVA with repeated measures on the subjective relevance ratings of the four decision factors (*substantial contract details, commitment*/*flexibility*, *personal experience*, and *secondary contract details)* and with cluster membership as the independent between-subject variable. Table 5 shows the results of the Bonferroni adjusted comparisons and the descriptive statistics. There were significant main effects for decision factors, *F*(3, 1080) = 428.84, *p* < 0.001, $\eta_{p}^{2}$ = 0.544, and cluster membership, *F*(2, 360) = 3.42, *p* = 0.034, $\eta_{p}^{2}$ = 0.019, as well as a significant interaction, *F*(6, 1080) = 13.13, *p* < 0.001, $\eta_{p}^{2}$ = 0.068. Pairwise comparisons indicated that the four decision factors were perceived to be of different relevance with regard to the decision at hand (*p* < 0.05 for all pairwise comparisons). In line with our definition of decision quality, consumers perceived substantial contract details (*M* = 5.10, *SD* = 0.69) as most important, followed by details on commitment/flexibility (*M* = 3.69, *SD* = 1.17), secondary contract details (*M* = 3.14, *SD* = 1.10), and personal experience (*M* = 2.98, *SD* = 1.18). More interestingly, we found that low effort-low focus consumers (*M* = 4.87, *SD* = 0.76) attributed less relevance to substantial contract details than either moderate effort-high focus (*M* = 5.29, *SD* = 0.55) or high effort-low focus consumers (*M* = 5.18, *SD* = 0.63). The latter two did not differ significantly. Instead, low effort-low focus consumers paid more attention to personal experience (*M* = 3.42, *SD* = 1.20) and secondary contract details (*M* = 3.77, *SD* = 1.15) than did participants with moderate effort and high focus (personal experience: *M* = 2.67, *SD* = 1.03; secondary contract details: *M* = 2.84, *SD* = 1.00) or participants with high effort and low focus (personal experience: *M* = 2.78, *SD* = 1.03; secondary contracts details: *M* = 3.20, *SD* = 1.09). The latter two differed with regard to the subjective relevance of secondary contract details, but not with regard to personal experience. No significant differences were found for details on commitment/flexibility. Figure 3 shows differences across decision factors as a function of cluster membership. Taken together, these findings reveal a coherent set of inter-cluster differences in the decision process. The low effort-low focus group did express no interest in substantial contract details (e.g., monthly charge), but was rather interested in questions of personal experience and secondary contract details (e.g., technical availability).

**TABLE 5:** Means, Standard Deviations and Results of the MANOVA on the Subjective Relevance of Contract Characteristics.

**Measure**	**low effort-low focus (*n* = 137)**		**moderate effort-high focus (*n* = 124)**		**high effort-low focus (*n* = 102)**		***F***	***p***	**$\eta_{p}^{2}$**
	***M***	***SD***	***M***	***SD***	***M***	***SD***			
Basic tariff details	4.87^a^	0.76	5.29^b^	0.55	5.18^b^	0.63	14.76	0.000	0.076
Commitment/flexibility	3.71	1.15	3.70	1.15	3.65	1.21	0.064	0.938	0.000
Personal experience	3.42^a^	1.20	2.67^b^	1.03	2.78^b^	1.03	16.75	0.000	0.085
Other tariff details	3.77^a^	1.15	2.84^b^	1.00	3.20^ac^	1.09	8.28	0.000	0.044

*Superscript letters indicate results of pairwise comparisons, with same letters indicating non-significant differences based on *p* < 0.05.*

### Information Processing and Decision Quality

Having found different information processing strategies across three clusters of participants, we turned to the question whether these differences in information processing behavior are associated with the quality of the final contract choice. Indeed, there were significant inter-cluster differences in decision quality, χ^2^(2) = 104.01, *p* < 0.001 (by Kruskal-Wallis test). Participants who had shown a medium amount of information processing with additionally higher focus on the best options (i.e., cluster: moderate effort-high focus) made decisions of higher quality (Mean Rank *MR* = 248.9) than participants who had shown a low amount of information processing with less focus on the best options (cluster: low effort-low focus; *MR* = 135.0), *Z* = 10.01, *p* < 0.001, and also than participants who had shown a high amount of information processing also not focusing on the best options (cluster: high effort-low focus; *MR* = 163.7), *Z* = 7.96, *p* < 0.001 (Mann-Whitney tests). Moreover, high effort-low focus participants reached a higher decision quality than did participants with low effort-low focus consumers, *Z* = 2.50, *p* = 0.012.

### Information Processing and Situational and Psychological Variables

Finally, we were interested in how the clusters differed on the individual and situational characteristics that were not included in the cluster analysis. Several univariate ANOVAs were calculated with cluster membership as the independent variables. Again, we used Bonferroni corrections to account or multiple comparisons. Table 6 gives an overview of the relationships across these additional measures. Table 7 shows descriptive statistics for the additional measures as well as the results of the comparisons.

**TABLE 6:** Correlation Coefficients.

**Measure**		**2.**	**3.**	**4.**	**5.**	**6.**
1.	Epistemic motivation	0.419**	−0.218**	−0.262**	0.393**	0.090
2.	Involvement		−0.464**	−0.372**	0.586**	–0.091
3.	Perceived decision difficulty			0.580**	−0.434**	–0.070
4.	Time pressure				−0.642**	0.014
5.	Satisfaction with decision					0.077
6.	Decision quality					

*Note:* Correlations reported on decision quality represent Spearman’s rho coefficients; **p < 0.01

**TABLE 7:** Means, Standard Deviations and Results of the ANOVA’s on the Additional Psychological Variables.

**Measure**	**low effort-low focus (*n* = 137)**		**moderate effort-high focus (*n* = 124)**		**high effort-low focus (*n* = 102)**		***F***	***p***	**$\eta_{p}^{2}$**
	***M***	***SD***	***M***	***SD***	***M***	***SD***			
Epistemic motivation	4.92	0.86	5.10	0.84	5.13	0.87	2.11	0.123	0.012
Individual involvement	4.51	0.71	4.48	0.63	4.34	0.67	2.04	0.131	0.011
Decision difficulty	1.80^a^	0.96	2.02^ab^	1.27	2.30^*b*^	1.37	5.16	0.006	0.028
Time pressure	1.80^a^	1.01.	2.03^a^	1.12	2.90^b^	1.40	27.90	0.000	0.134
Satisfaction	5.12^a^	0.68	5.19^a^	0.67	4.82^b^	0.85	8.19	0.000	0.043

*Superscript letters indicate results of pairwise comparisons, with same letters indicating non-significant differences based on *p* < 0.05.*

The three information processing clusters did not differ significantly on epistemic motivation, *F*(2, 360) = 2.11, *p* = 0.123, or involvement with the decision, *F*(2, 360) = 2.04, *p* = 0.131. However, the analysis of both perceived decision difficulty, *F*(2, 360) = 5.16, *p* = 0.006, $\eta_{p}^{2}$ = 0.028, and perceived time pressure, *F*(2, 360) = 27.90, *p* < 0.001, $\eta_{p}^{2}$ = 0.134, revealed significant differences between clusters. High effort-low focus consumers found the decision to be more difficult (*M* = 2.30, *SD* = 1.37) than low effort-low focus consumers (*M* = 1.80, *SD* = 0.96). The remaining comparisons were not significant. Similarly, high effort-low focus consumers reported greater time pressure (*M* = 2.90, *SD* = 1.40) than did low effort-low focus (*M* = 1.80, *SD* = 1.01) or moderate effort-high focus consumers (*M* = 2.03, *SD* = 1.12). The latter two clusters did not differ significantly from each other. Finally, given the differences in decision quality, we asked whether clusters differed in their satisfaction with their decision. Satisfaction was significantly lower among high effort-low focus participants (*M* = 4.82, *SD* = 0.85) than among low effort-low focus participants (*M* = 5.12, *SD* = 0.68) and moderate effort-high focus participants (*M* = 5.19, *SD* = 0.67); the latter did not differ significantly from each other, *F*(2, 360) = 8.19, *p* < 0.001, $\eta_{p}^{2}$ = 0.043 (see Table 7 for descriptive statistics and results of the comparisons).

## Discussion

The present study identified three types of participant (i.e., would-be consumers) with distinctive information processing styles and showed the relevance of these styles for optimal decision-making in a consumer choice task.

Using information processing data obtained with tracking software, we identified three distinct clusters with a high degree of accuracy. Our results suggest that all behavioral tracking variables entered into cluster analysis significantly contributed to the segmentation. However, the time parameters (time and portion of time) and the comparison index were most successful in predicting cluster membership. As expected, the clusters were arrayed along two dimensions – the *extent* dimension, referring to the overall effort someone puts into the information processing, and the *focus* dimension, referring to the degree someone is able to correctly focus onto the best available options. A certain combination of these two dimensions determines whether one’s information processing reflects a more systematic (i.e., moderate effort, high focus) or less systematic (i.e., low effort, low focus) mode of processing. Three subgroups, each with a distinct profile along these two dimensions, can be summarized as follows: (1) participants with a low-effort and low-focus information processing strategy (*n* = 137); (2) participants with a moderate-effort and high-focus information processing strategy (*n* = 124); and (3) participants with a high-effort and low-focus information processing strategy (*n* = 102).

Low effort-low focus consumers showed the lowest scores on both the extent and the focus of information processing. Participants in this cluster engaged in a fast and intuitive decision-making mode. Although we did not use measures on the exact strategies employed during information processing, the ratings on the subjective importance of 25 different contract characteristics indicated that participants in this cluster based their decisions mainly on previous experience or gut feelings, while paying less attention to the most important substantial contract details. Following Delaney et al. (2015), we refer to these participants *“affective experientialists.”* High effort-low focus consumers at the other end of the spectrum displayed the most extensive information search, but were, similar to low effort-low focus consumers, unable to correctly focus onto the optimal contracts. When also considering the subjective relevance ratings, it becomes evident that although this group of participants (correctly) perceived substantial contract details as being most important, they were not able to filter out less relevant (with regard to our definition of decision quality) secondary contract details. This cluster represents the systematic mode of processing as participants tried to process all information available and integrate it into their decision. However, due the amount of information available, information overload may have arisen. Although we did not measure information overload directly, we found that participants in this cluster perceived greater time pressure, higher decision difficulty, and were less satisfied with their decision. These measures may serve as proxies for information overload (Hahn et al., 1992; Iyengar and Lepper, 2000; Carmon et al., 2003; Lee and Lee, 2004; Paul and Nazareth, 2010). Accordingly, we refer to the participants in this cluster as *“confused perfectionists.”* Finally, we found a third cluster between these two end-point segments in that the moderate effort-high focus consumers were best able to focus on the optimal contract while only investing a moderate amount of effort. Additionally, the subjective relevance ratings showed that these participants paid close attention to the most important substantive contract details, while only skimming peripheral details. Accordingly, we refer to participants in this cluster as *“ecological rationalists”* (cf. Kasper et al., 2010).

In line with previous research on consumer segmentation (e.g., Kasper et al., 2010; Delaney et al., 2015; Mittal, 2017), our results correspond to the basic dichotomy suggested by dual-processing models (e.g., Evans, 2008); yet, they include another cluster of consumers that ranges between the two end-points. Among previous attempts to segment consumers, the study of Mittal (2017) is most comparable in terms of the underlying concept of decision making. Mittal found four segments that differ along two basic traits – extent (extending versus info-mising) and goal (optimizing versus satisficing) of information processing. When considering the structural meaning of the segments, there is some overlap with the results of our study. Mittal found a segment of *optimizing extenders* which corresponds to our second cluster of *confused perfectionists*. These individuals strive for exhaustive information processing. However, consumer confusion as seen among some participants in our study environment made it impossible to find the optimal decision with such an exhaustive strategy. On the other end of the spectrum, Mittal (2017) identified a segment of *confused, unwilling*, i.e., individuals that do not care about getting the best solution and a segment of *snap deciders*, i.e., individuals that invest minimal effort and decide almost solely based on their gut feelings. Both these segments fall within our cluster of *affective experientialists* whose participants invested only minimal effort and based their decision mainly on previous experience or gut feelings. Finally, we (*ecological rationalists)* and Mittal (*balanced diligent)* found a segment of consumers, who consider an adequate amount of information without being too extensive in their information processing.

Our final cluster solution also shows some common clusters with those of other studies, for instance Delaney et al. (2015) and Makgosa and Sangodoyin (2018). However, differences in sampling, measurement instruments, and analytical strategy may be responsible for differences in the exact number of segments identified. Most importantly, consumer segmentation studies are typically based on survey questionnaires that ask participants to indicate their typical shopping behavior. In contrast, we obtained segmentation from behavioral measures in a high information load environment. As a consequence, the comparability across studies is limited. For instance, in a typical shopping scenario that is not characterized by high information load, we would have expected to find a cluster of individuals with high effort while at the same time having a high focus. Given the complexity of the task and the amount of information available in our study, individuals may have been simply not able to maintain a high focus with such an extensive processing strategy.

The central finding of this research is that participants who may be described as ecological rationalists were not only able to focus on the three best options but rather compare them more frequently with each other. It seems that only this kind of systematic behavior leads then to the objectively best choices. Both the confused perfectionists and affective experientialists were less successful. In line with the framework of ecological rationality (Gigerenzer and Brighton, 2009), successful individuals appeared to reduce information complexity while keeping decision quality at satisfactory levels (Kasper et al., 2010). Given the complexity of the task and the amount of information available, this seems to be a reasonable and resource-conserving approach. Even more important to policy makers, there is a large group of individuals who did not arrive at optimal decisions, but for different reasons. Faced with a complex decision and high information load, the confused perfectionists failed to find an adequate decision-making strategy (Hwang and Lin, 1999; Sasaki et al., 2011). Conversely, the affective experientialists went too far when seeking to reduce information complexity. Whereas ignoring parts of the available information can be an effective and efficient strategy to cope with high information load (Eppler and Mengis, 2008; Krueger et al., 2020), ignoring too much information compromises decision quality. Thus, neither the exhaustive use of information, nor the use of very little information yielded optimal decisions. In order to *effectively* use the information provided, one has to identify and correctly focus on the most important pieces of information and bring them together into a choice.

Previous research found that whether individuals are willing to engage in effortful processing depends on their epistemic motivation (Scholten et al., 2007), but in the present study, we found no significant differences in *epistemic motivation* and the involvement with the decision between clusters. Similarly, the willingness to engage in deliberate information processing is typically associated with the level *of personal involvement*. Highly involved individuals tend to put more effort into making a thoughtful decision, take more time, and show higher cognitive activity in order to reduce the risk of a false decision. By contrast, individuals with little involvement tend to use simpler information processing strategies and are more likely to rely on peripheral cues (Petty and Cacioppo, 1984; Hansen, 2005). Although we cannot draw causal inferences from the available data, we offer some speculations. Over many years of repeated practice of the same strategy in a multitude of product choices, individual information processing strategies may have become habitual, and thus are not so much subject to situational characteristics, such as motivation or involvement (Mittal, 2017). Future research should address this issue by, for instance, experimentally manipulating the level of involvement or epistemic motivation.

### Implications

The present research was motivated by the idea that the current market of cellular services does not give consumers enough guidance to make optimal choices. Particularly, when information load is high, it is difficult to optimize decision quality. Only few consumers are both able and willing to process all available information (Kasper et al., 2010), thus consumers remain at risk to make choices against their own best interests. Providers may take advantage of consumers’ limited capacity to process information and thereby exacerbate “behavioral market failures” (Lunn, 2013). Contrary to what is intended by consumer protection regulation, providing ever more information does not improve consumer decision-making. Most consumers find it difficult to extract the most relevant pieces of information. Poorly written or confusingly presented information invites consumers to “tune out” (Lange and Krahé, 2014). The challenge to regulators is to find ways to make consumer information more *effective*. Identifying and exploring different types of consumers may be a promising approach to consumer protection. With information on different consumer segments, legislation should seek to support those consumers whose ability to distinguish between “relevant” and “less relevant” information is limited. A consumer-oriented legal environment would facilitate the search for suitable contracts without requiring a review of excessive amounts of information and without making unreasonable demands on consumers’ time. Thus, information regulation should not aim at the one-dimensional delivery of a certain content but rather at providing guidance on how to deal with the information successfully. This aim may be achieved with decision aids designed to spare consumers mental effort. For example, information can be presented in a way that provides a comprehensive picture of the most essential attributes of a certain product or service, or that increases the comparability across similar products or services both in terms of content as well as in terms of organization and design. Another option is to use computer-based technologies to assist individuals in making decisions and to align the search process with the individual preferences (e.g., use patterns) of the consumers (Lee and Cho, 2005). The difficulties of consumers to cope with the extraordinary high load of information cannot only be tackled by regulation of the relationship between the contract parties, but also by more market oriented regulatory tools, as, for example, reliable total-cost calculators based on up-to-date data on the offers of all providers in the market. Though some of these instruments, such as calculators or search engines, already exist, empirical data show that consumers do not make adequate use of them or do not trust in the results provided by these instruments (Dinslaken et al., 2020). Thus, legislation should aim to increase use and acceptance of such instruments. Future studies testing the effects of enhanced information design and electronic and other decision aids on decision quality could form the basis for the development of more effective or “smarter” regulatory environments that lead consumers to a higher percentage of good contract choices than the present environment.

### Limitations

Some limitations of the present study should be noted. We focused on one market in one country, which limits the generalizability of the results. However, within the consumer segmentation literature, consumer decision-making is typically viewed as a mental orientation that reflects a consumer’s approach to making product choices (Sproles and Kendall, 1986). Likewise, Schwartz et al. (2002) differentiate between ‘maximizing’ and ‘satisficing’ personalities of consumers characterizing a consumer’s trait-like approach to making choices. Thus, decision-making styles are thought to persist across different goods and services as they reflect a personality trait. There is also a considerable amount of overlap in the structural meaning of clusters across different segmentation studies, including ours, which leaves us optimistic with regard to the validity of our results. To further validate our results, the segmentation should be verified for other types of service market and countries with larger and more representative samples using additional clustering variables. Finally, the choice of clustering variables, is, in part, subjective. Thus, employing other clustering variables may result in a different segmentations.

Critically, there is a conceptual overlap in the operationalization of our focus measures, i.e., clicks and time spent on the three best options and decision quality, i.e., actually deciding on one of the tree best options (or any other option). As a consequence, focus and decision quality are confounded as focus is part of the segmentation and also a proxy for decision quality itself. However, the correlation between both measures is not perfect (correlation between decision quality and time *r* = 0.603, and clicks on three best *r* = 0.710) indicating that although some individuals correctly focused on the three best options during information search, they decided on something completely different.

Our method did not afford precise inferences about participants’ individual information processing strategy. Although we went beyond previous research by using behavioral measures, we had no direct access to participants’ specific decision strategies or heuristics. Our measures were proxies. It is possible that at any stage of the search process participants used a certain unmeasured strategy that helped them to choose the most relevant pieces of information. As a consequence, we do not know if participants in the low-effort and low-focus cluster truly relied on heuristics in their decisions or whether they were simply less systematic because they wanted to get through the task.

Finally, we did not measure the extent to which individuals were affected by information load directly. Based on previous research (Turnbull et al., 2000; Kasper et al., 2010), we assume that our method did create information overload, but future studies should measure load directly. With the use of self-report measures of information processing, such as the need for additional information (Chaiken et al., 1989), perceived consumer confusion (Kasper et al., 2010), or perceived information gathering capacity (Kahlor et al., 2003), it is possible to explore the relationship among information load and information processing behavior more deeply.

## Conclusion

Based on actual information processing data obtained with tracking software, three consumer segments were identified that differed not only in their information processing strategy but also in their ability to make optimal decisions. We found that individuals facing a complex and real-life consumer decision characterized by high information and choice load were most successful by applying information processing strategies that involved the focused selection and processing of a medium amount of information. Processing strategies involving only very small amounts of information, as well as strategies requiring a very extensive analysis of the available information, were found to be less successful. By enhancing the salience, simplicity and clarity of relevant contract related information, together with providing software devices that simplify information processing, consumer regulation could particularly support those individuals who suffer from the high information and choice load.

## Data Availability Statement

The datasets presented in this study can be found in online repositories. The names of the repository/repositories and accession number(s) can be found below: https://osf.io/cbp2t/?view_only=793b1ccfb58643d98e55291557c53103.

## Ethics Statement

The studies involving human participants were reviewed and approved by Ethikkommission, Universitätsplatz 3, 8010 Graz. Written informed consent for participation was not required for this study in accordance with the national legislation and the institutional requirements. All participants gave their written informed consent.

## Author Contributions

CV-H did the concept and design of the project, data analyses and interpretation, and writing the manuscript. ID and FC did the concept and design of the project and data acquisition. JK did the concept and design of the project and editing of the manuscript. JA did the concept and design of the project and data acquisitions and preparation. AF did the concept and design of the project. HB did the checking the manuscript format and data visualization. UA did the concept and design of the project, supervision of the development, and editorial of the manuscripts. All authors contributed to the article and approved the submitted version.

## Conflict of Interest

The authors declare that the research was conducted in the absence of any commercial or financial relationships that could be construed as a potential conflict of interest.

## Footnotes

1 Supplementary material, which is not part of the paper, as well as data and analysis script are available at the Open Science Framework via https://osf.io/cbp2t/?view_only=793b1ccfb58643d98e55291557c53103.

## Supplementary Material

Supplementary Images 1 and 2 View of web-platform with hyperlinks to all cellular service contract options available.

Supplementary Image 3 Dendrogram using Ward Linkage.

Supplementary Table 1 Frequency statistics of decision quality.
