# Supplementary figures and images for: Not Too Much and Not Too Little: Information Processing for a Good Purchase Decision
Source: Front Psychol. 2021 Apr 28;12:642641. doi: 10.3389/fpsyg.2021.642641 (PMC8115021; doi:10.3389/fpsyg.2021.642641)

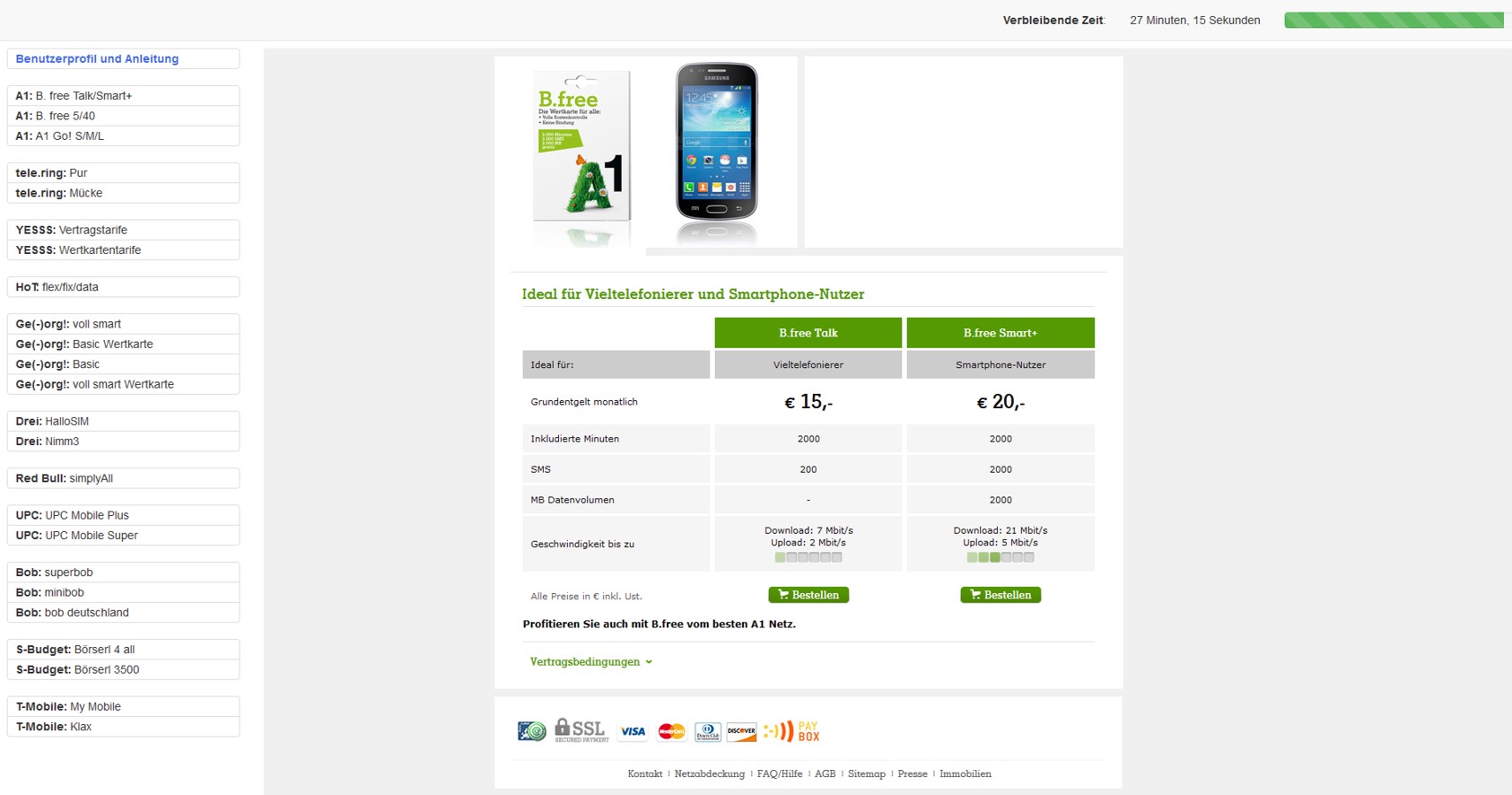

Supplement: Supplementary file 1 [file Image_1.jpg]

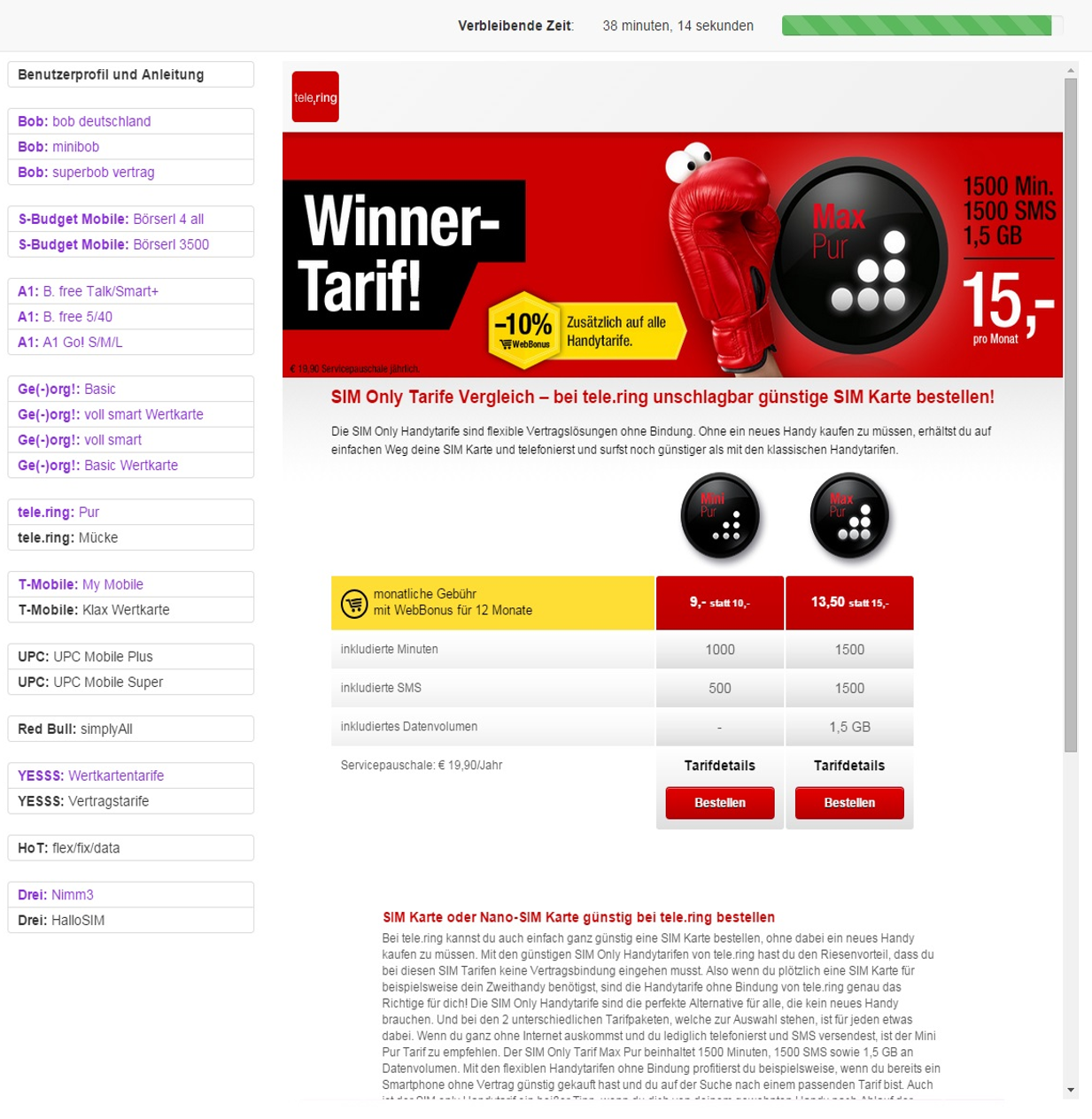

Supplement: Supplementary Images 1 and 2 — View of web-platform with hyperlinks to all cellular service contract options available. [file Image_2.png]

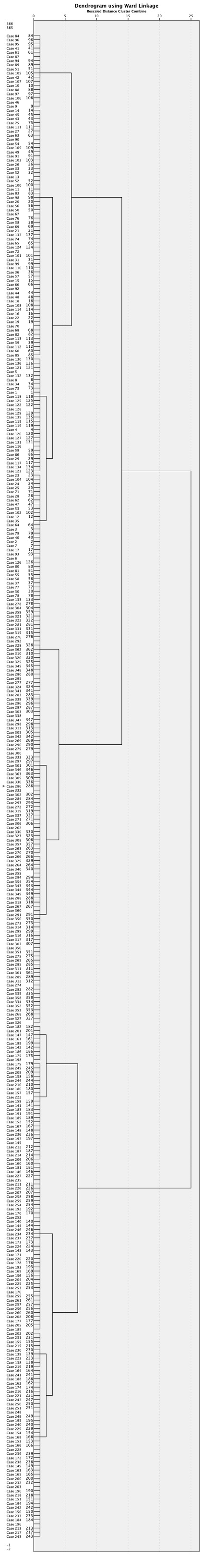

Supplement: Supplementary Image 3 — Dendrogram using Ward Linkage. [file Image_3.jpg]
