# Supplementary material for: Not Too Much and Not Too Little: Information Processing for a Good Purchase Decision
Source: Front Psychol. 2021 Apr 28;12:642641. doi: 10.3389/fpsyg.2021.642641 (PMC8115021; doi:10.3389/fpsyg.2021.642641)
Supplement: Supplementary Table 1 — Frequency statistics of decision quality. [file Table_1.docx]

Supplementary Table 1: Frequency statistics of decision quality

| Rank | *n* | % | Monthly costs | Decision quality |
| --- | --- | --- | --- | --- |
| 1 | 207 | 57 | 14.90 | 4 |
| 2 | 63 | 17.4 | 19.67 | 3 |
| 3 | 19 | 5.2 | 20.00 | 3 |
| 4 | 8 | 2.2 | 20.74 | 2 |
| 5 | 3 | 0.8 | 21.66 | 2 |
| 6 | 7 | 1.9 | 22.78 | 2 |
| 7 | 1 | 0.3 | 24.66 | 2 |
| 8 | 6 | 1.7 | 25.67 | 1 |
| 9 | 3 | 0.8 | 31.66 | 1 |
| 10 | 1 | 0.3 | 33.23 | 1 |
| 11 | 1 | 0.3 | 38.02 | 1 |
| 12 | 2 | 0.6 | 41.66 | 1 |
| 13 | 4 | 1.1 | 42.71 | 1 |
| 14 | 7 | 1.9 | 49.02 | 1 |
| 15 | 3 | 0.8 | 51.71 | 1 |
| 16 | 1 | 0.3 | 57.06 | 1 |
| 17 | 3 | 0.8 | 60.46 | 1 |
| 18 | 1 | 0.3 | 80.48 | 1 |
| 19 | 1 | 0.3 | 83.60 | 1 |
| 20 | 3 | 0.8 | 88.14 | 1 |
| 21 | 1 | 0.3 | 89.55 | 1 |
| 22 | 1 | 0.3 | 89.63 | 1 |
| 23 | 3 | 0.8 | 104.05 | 1 |
| 24 | 1 | 0.3 | 142.25 | 1 |
| 25 | 1 | 0.3 | 158.05 | 1 |
| 26 | 1 | 0.3 | 162.69 | 1 |
| 27 | 2 | 0.6 | 261.38 | 1 |
| 28 | 1 | 0.3 | 296.04 | 1 |
| 29 | 2 | 0.6 | 476.60 | 1 |
| 30 | 2 | 0.6 | 644.65 | 1 |
| 31 | 2 | 0.6 | 1171.00 | 1 |
| 32 | 1 | 0.3 | 1338.65 | 1 |
| 33 | 1 | 0.3 | 3945.96 | 1 |
